# A framework for assessing the feasibility of malaria elimination

**DOI:** 10.1186/1475-2875-9-322

**Published:** 2010-11-11

**Authors:** Bruno Moonen, Justin M Cohen, Andy J Tatem, Jessica Cohen, Simon I Hay, Oliver Sabot, David L Smith

**Affiliations:** 1 Clinton Health Access Initiative, Boston, USA; 2 Global Health Group, University of California, San Francisco, California, USA; 3 Emerging Pathogens Institute, University of Florida, Gainesville, USA; 4 Department of Geography, University of Florida, Gainesville, USA; 5 Harvard School of Public Health, Harvard University, Boston, MA, USA; 6 Spatial Ecology and Epidemiology Group, Tinbergen Building, Department of Zoology, University of Oxford, South Parks Road, Oxford, UK; 7 Department of Biology, University of Florida, Gainesville, USA

## Abstract

The recent scale-up of malaria interventions, the ensuing reductions in the malaria burden, and reinvigorated discussions about global eradication have led many countries to consider malaria elimination as an alternative to maintaining control measures indefinitely. Evidence-based guidance to help countries weigh their options is thus urgently needed. A quantitative feasibility assessment that balances the epidemiological situation in a region, the strength of the public health system, the resource constraints, and the status of malaria control in neighboring areas can serve as the basis for robust, long-term strategic planning. Such a malaria elimination feasibility assessment was recently prepared for the Minister of Health in Zanzibar. Based on the Zanzibar experience, a framework is proposed along three axes that assess the technical requirements to achieve and maintain elimination, the operational capacity of the malaria programme and the public health system to meet those requirements, and the feasibility of funding the necessary programmes over time. Key quantitative and qualitative metrics related to each component of the assessment are described here along with the process of collecting data and interpreting the results. Although further field testing, validation, and methodological improvements will be required to ensure applicability in different epidemiological settings, the result is a flexible, rational methodology for weighing different strategic options that can be applied in a variety of contexts to establish data-driven strategic plans.

## Background

During the Global Malaria Eradication Programme (GMEP) and the decade that followed, 37 countries with endemic malaria succeeded in eliminating transmission [1]. Since 1978, however, only nine more countries have reached this goal, while most malaria-endemic countries have aimed for control, not elimination [2]. Recently, some of the countries that halved their malaria cases between 2000 and 2008 [1] are revising their strategic plans and are considering elimination as an alternative to maintaining control measures indefinitely. In the wake of Bill and Melinda Gates' 2007 commitment to global malaria eradication [3,4], officials from many countries where elimination was considered infeasible during the GMEP because of high endemicity and poor infrastructure [5-7], including Nigeria [8], Ghana [9], Tanzania [10], and Kenya [11], have announced plans to eliminate malaria. It has been argued that premature commitment to elimination may be counterproductive as it could divert limited resources and negatively impact efforts to reduce the high burden of malaria [3,12,13]. There is thus an urgent need for clear, evidence-based guidance to assess whether elimination represents a realistic goal in a given region.

Weighing elimination today is different from the beginning of the GMEP era, when the impact of eradication programs essentially was taken for granted. The spectacular early success of the GMEP in Europe [5,6,14,15] was not replicated elsewhere, however, and the early timelines and cost estimates proved overly optimistic [7]. The GMEP adapted by recommending that countries assess the feasibility of such an undertaking through "a preliminary study to accumulate and analyze the information required for realistic programme planning" before embarking on a costly and potentially ineffective campaign [16,17]. These studies were intended to "cover not only technical and operational aspects but also a wider sphere, with a view to elucidating the socio-economic implications of malaria eradication within the context of the overall development plan of the country and its human and financial resources" [14]. Current World Health Organization (WHO) guidelines continue to recommend assessing the feasibility of elimination, but they only propose a qualitative assessment using a checklist of technical and operational pre-conditions and provide no guidance on how to quantitatively assess key epidemiological factors that define the feasibility of achieving and maintaining elimination [18].

**Figure 1:**
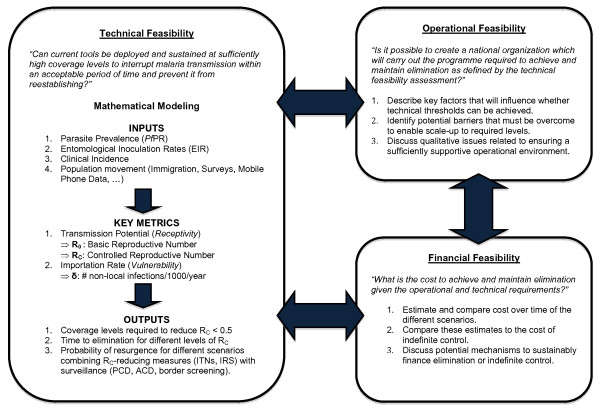
**Framework for assessing the feasibility of malaria elimination**. Technical feasibility identifies requirements for achieving and maintaining elimination under different scenarios, operational feasibility involves the administrative and programmatic capacity to meet those requirements, and financial feasibility examines the costs involved. Unrealistic operational expectations and/or prohibitively high costs will require considering other technically feasible scenarios that can be achieved given operational or financial limitations.

In this paper, a framework for assessing the feasibility of elimination in a given region is described that is based on literature accumulated during the GMEP and updated by the experience of conducting the first contemporary malaria elimination feasibility assessment in Zanzibar [19]. This framework, and the methods proposed, will need to be further evaluated and adapted as necessary to ensure its suitability in different eco-epidemiological settings. The goal is to provide malaria programmes with the basis for a practical blueprint for making an evidence-based decision. Unlike the GMEP, eliminating countries today cannot expect their neighbors to eliminate malaria, so they must consider requirements both for interrupting transmission and remaining malaria-free despite continued importation of infections [20]. The proposed framework is comprised of three axes: the technical requirements to achieve and maintain elimination, the operational capacity of the malaria programme and the public health system to meet those requirements, and the financial feasibility of funding the necessary programmes over time (Figure 1). However, this paper prioritizes technical feasibility as no quantitative metrics have previously been offered for its assessment while previous discussions of operational [18,20,21] and financial feasibility [22-24] are more extensive. The ultimate goal of this exercise is to educate decision-makers about their options and the likely consequences of their decisions.

## Framework

### Technical feasibility

Elimination is technically feasible if current tools can be deployed and sustained at sufficiently high coverage levels to interrupt malaria transmission within an acceptable period of time and prevent it from reestablishing [25]. Current WHO guidance avoids directly evaluating technical feasibility by instead stating that a prerequisite for an elimination attempt is "demonstrated technical feasibility of malaria elimination in similar eco-epidemiological settings in the recent past" [18]. However, the fact that elimination has not been achieved in sub-Saharan Africa means another approach to assess its feasibility is required. Here, the updated technical feasibility assessment is based on an assembly of data sources and a stratification of risk across the region of interest according to key metrics related to local transmission potential and the importation of infections from the outside. Mathematical models are then used to estimate the time to eliminate malaria in relation to transmission reductions that are expected at various intervention effective coverage levels. The same metrics also inform planning for a post-elimination transition and programmatic reorientation to remain malaria free.

#### Key metrics

To assess the technical feasibility of achieving and maintaining elimination in a particular region, two key factors must be assessed: the *transmission potential *and the *importation rate *[26]. These two quantities, historically also called "receptivity" and "vulnerability", interact to comprise the *malariogenic potential*, or overall malaria risk, and are defined in Appendix 1.

**Figure 2:**
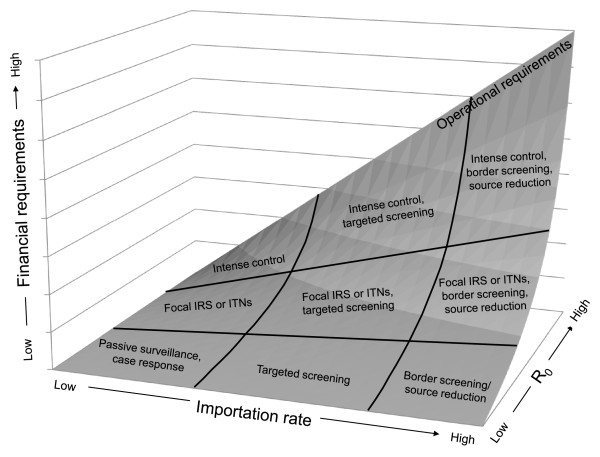
**Relationship between technical, operational, and financial components of the feasibility assessment**. Technical feasibility is determined by the importation rate and intrinsic transmission potential, R_0_, operational feasibility is determined by the required degree of interventions needed to achieve elimination given these technical metrics, and financial feasibility depends upon the government's ability to secure sufficient funds to maintain the required operational measures.

In general, a higher transmission potential will require higher coverage levels to interrupt transmission and control outbreaks, while a greater importation rate will set expectations about the frequency of outbreaks and resources required to prevent reemergence. For example, if transmission potential is very low, a modest level of control measures and modest costs may be sufficient to halt endemic transmission regardless of the importation rate; such situations resemble Europe and other places where the GMEP was successful. If importation and transmission potential are each very large, however, the technical requirements for achieving elimination may be prohibitively high, given the operational constraints and the financial requirements (Figure 2). Although WHO guidelines emphasize the importance of these factors [18,27], specific instruction on how they should be quantified has never been given. Accordingly, the following specific metrics to quantify each of these key factors are proposed below.

#### Transmission potential metrics

Maximum, or "intrinsic", transmission potential is ideally measured at the pre-control level, and it is represented by the epidemiological parameter R_0_, the basic reproductive number [28-30]. R_0 _describes the number of human malaria cases that would arise from a single malaria case if all control measures were removed and if malaria immunity had completely waned. It therefore determines the rate of increase in the number of cases during a malaria epidemic over time, or alternatively, the proportional reductions in transmission required to eliminate malaria from its pre-control level [31,32]. R_0 _in any population is determined by a complex combination of factors that describe how hospitable a particular region is for malaria, including the ecology and efficiency of mosquito vector species [33], and the socioeconomic characteristics of its inhabitants [14,28,34]. R_0 _is also important for post-elimination planning since it describes the magnitude of potential resurgence if interventions are scaled back.

Most populations are now under some form of malaria control. Under control, transmission potential is measured by the controlled reproductive number, called R_C_. This number describes the number of infections that each infected person will generate under a given level of control activities and given immunity [35], and is always smaller than the intrinsic potential. Transmission potential can be rapidly reduced from its intrinsic baseline through vector control interventions and prompt and effective treatment [36-38]. If R_C _is less than one, then malaria will be eliminated on timelines that depend, to a large extent, on the value of R_C _[39]. Importantly, both R_0 _and R_C _can change over time. Changes in transmission potential are attributed to R_C _if they involve changes in insecticide-treated net (ITN) ownership or usage, indoor residual spraying (IRS) coverage, the potency of the insecticides, or the way people access health clinics. Changes in R_0 _occur if they would have happened regardless of interventions, such as transformations in climate, development, or land-use.

These metrics can be inferred from one of several field measures of the intensity of transmission [28,29], albeit with uncertainty, including the parasite rate (PR) [40], the entomological inoculation rates (EIR) [41,42], or direct measures of vectorial capacity [43,44]. Measuring vectorial capacity directly is challenging and difficult to implement at scale, so in most cases R_0 _and R_C _must be calculated from PR or EIR using mathematical models [28,29]. Mathematical transmission models incorporating classical transmission parameters like the number of bites a human receives per day, the percent of bites that infect a mosquito, the duration of human infection, and the survival time of the mosquito can convert field-based measures of transmission into R_0 _or R_C _[28].

A first step of conducting a technical feasibility assessment thus will involve collating available malaria surveys from both the past and present. This information allows calculation of these transmission metrics and provides valuable insight into how transmission has changed over time and what can realistically be expected in the future. Historical PR or EIR surveys conducted before control measures were implemented can be used to estimate R_0_, unless the epidemiology has changed due to other factors. Many surveys appropriate for R_0 _calculation were conducted during GMEP, since a general epidemiological survey was considered to be the first step of an elimination programme [25]. Contemporary parasitological or entomological surveys at current levels of control measures can also be used to calculate R_C _[35], and by estimating the effect of existing intervention effective coverage levels, it is then possible to back-estimate R_0_. The presence of vector control measures will result in lowered vectorial capacity [43], with the magnitude of their influence dependent upon the effective coverage of interventions and the vector species present [45]. Some vectors feed or rest outdoors, so they are more difficult to control with existing vector interventions. Databases describing the local distribution of vector populations therefore will inform all aspects of planning [35].

#### Importation metrics

As long as current transmission potential (i.e., R_C_) is above zero, the importation of malaria parasites by infected individuals or mosquitoes will result in some local transmission; imported malaria and R_C _thus jointly influence the difficulty of reaching a defined end goal. An infection can be imported into a given region in one of four ways: 1) human residents acquire malaria while travelling outside the region, then return; 2) infected humans immigrate into the region (and, like residents, are thus likely to remain in the region throughout their infectious period); 3) infected humans travel through the region (and are thus likely to spend a shorter period of time in the region while infectious); or 4) infected mosquitoes migrate into the region. All these pathways lead to risk of locally-acquired infections, and so the importation rate should be summarized in terms of the number of infections that originated somewhere else, represented here as δ. Malaria importation rates are ideally expressed at an annual rate of incoming infections per 1,000 population. In many countries contemplating elimination (especially those sharing land-borders with highly endemic regions), as well as those who have eliminated, δ is likely to be quite high [46], and it will lead to low levels of malaria transmission unless R_C _= 0 [47]. As with transmission potential, the importation rate can change seasonally because of migration and transmission patterns in neighboring regions and annually because of instability or changes in tourism or immigration patterns.

Methods for calculating δ are less robust at present than those for R_0 _and R_C_, in part because importation has limited importance when endemic malaria is tolerated. Some methods were, however, developed to estimate δ for the islands of Zanzibar [19]. One method involves collecting data on the entry routes through which humans (or mosquitoes) travel into the region of interest and the number that travel through those routes, including roads, bus routes, and airplane traffic. New data sources, such as mobile phone usage data - a proxy measure for population movement - appear promising for the future, especially in areas where coverage and ownership are high [48]. Other methods or data are then required to estimate the fraction of those individuals infected with parasites and their contribution to onwards transmission. The risk of a resident from the region bringing home an infection from a visited endemic area, for example, can be calculated based upon the force of infection in the visited region (which can be identified from existing transmission maps [49]) as well as the time spent there. Imported malaria plays an increasingly important role in transmission as endemicity declines, so in low endemic areas, the travel histories of malaria cases can help to estimate δ as well as R_C _[47]. Robust approaches to estimating δ will continue to be improved by opportunities offered by combining geographical information systems, spatial statistics [49], and the increasing ability to quantify and evaluate the epidemiological impact of human transport systems [46,50,51].

#### Interpretation

Measures of the transmission potential and the importation rate provide the raw data for evaluating whether elimination should be deemed technically feasible or not. During the GMEP, such determination came in the form of observations regarding the success of pilot projects [25]. Over time, these observational approaches may again prove useful as experience in relevant settings dictates what values of R_0 _or importation are too high to permit successful elimination. Limited experience with elimination in most of the world, meanwhile, suggests that the historical precedent approach is inappropriate. Instead the technical feasibility of interrupting transmission and preventing reemergence may be simulated with mathematical models. These models require clearly defined endpoints to interpret the outcomes and to set thresholds for "failure" when running simulations. It has been argued that "zero local transmission" might not be the most appropriate operational definition for elimination and that different endpoints will need to be defined [47].

#### Is it technically possible to achieve elimination?

**Figure 3:**
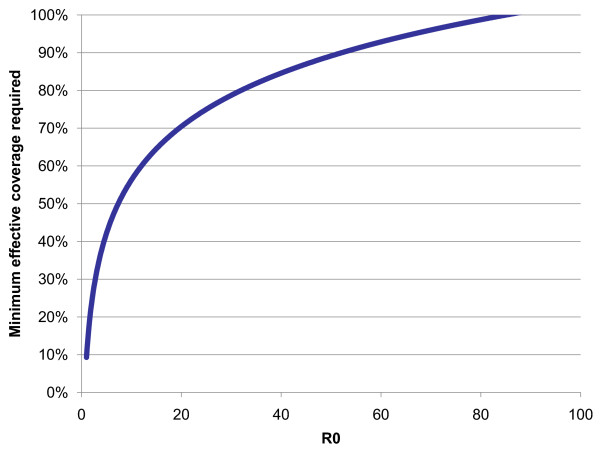
**The effort required to reduce R_C _below 1 (in terms of fraction of the population effectively covered by protection measures like IRS or ITNs) increases with greater intrinsic transmission potential**.

Endemic malaria transmission will eventually cease as long as R_C _is reduced to <1 throughout the region, although for elimination within a reasonable timeframe, it will be necessary to reduce it to below 0.5 [39]; projected timelines to elimination depend on population size and many other factors, but simulations suggest that elimination would take approximately 12.6 years in a population of a million if R_C _were 0.5 compared to 25.2 years if it were 0.75 [39]. The higher the intrinsic transmission potential, the greater the reduction in transmission required to depress it to these levels. This can be done by increasing coverage intensity and/or usage of existing interventions, or by deploying additional interventions. At sufficiently high potential, even total coverage with all currently available interventions may fail to achieve elimination; in other words, elimination is technically infeasible at those levels. Figure 3 depicts how the fraction of the population that must be completely protected by ITNs for elimination increases with R_0_, according to one recently published mathematical model [35] (other models may produce different but analogous curves).

The effective coverage levels depicted in Figure 3 may correspond to any intervention that results in the protection of individuals from infection, such as ITNs, IRS, or (when it is available) a vaccine. Future research will be necessary to differentiate the effects of different interventions or combinations thereof. Although the effects of control measures that act on different components of the transmission pathway, such as larval control, ITNs, or anti-malarial drugs, are known to combine their effect multiplicatively [52], it is less clear how the effect of different interventions acting on the same component, like ITNs and IRS, varies; additional questions surround how that effect changes when they are used in combination. Recent research indicates that there may be an additional protective effect when combining IRS with ITNs [53], but more investigation is needed.

Projected timelines to elimination must come with a set of caveats as existing data may provide only limited information about whether R_C _is sufficiently low throughout the region of interest; for example, local foci in which R_C _remains very high are likely to occur and to require particular attention [21]. Notably, actual timelines for elimination will depend on the ability to deal with these foci; an initial assessment of timelines can be modified as it becomes possible to stratify risk at higher spatial resolution. In these foci, it will be necessary to intensify control measures, while maintaining minimum effective coverage levels elsewhere to keep R_C _< 1. This important threshold determines if malaria transmission is decreasing or increasing within the region, and thus provides vital information on the minimal required capabilities of the elimination programme that will set the standards for assessing operational and financial feasibility.

#### Is it technically possible to remain malaria-free?

Assuming it is determined to be technically feasible to eliminate malaria, the next question is whether it is technically possible to prevent its reemergence. Clearly, it is theoretically possible to remain malaria-free by simply maintaining the same high effective coverage levels that achieved elimination, but it is highly unlikely that this would be cost-effective compared with the lower effective coverage levels and comparable benefits of achieving controlled low-endemic malaria (CLM). Instead, an optimum package of interventions required to maintain elimination should be identified and evaluated, taking into account the rate at which infections are imported, δ, and the probability of each imported infection leading to onwards transmission, R_C_. A simple consideration of the probability of a given level of local transmission occurring given δ and R_C _is possible using branching processes [47], and maximum values for the two parameters can be calculated that yield what is deemed to be an acceptable risk of a given level of transmission (e.g., a goal of maintaining <1% risk of 5 or more cases occurring in a given year).

Although branching processes describe the strength of post-elimination control measures required to keep malaria occurrence at acceptable levels, such simple models yield little precise information with which to assess operational and financial feasibility. A more robust and flexible assessment involves simulation models of outbreak and control to evaluate specific protocols and the potential of preventing reemergence given the joint risk of importation and transmission. It is thus recommended that simulation models be used to make predictions about the risk of malaria reemergence following elimination given specified levels of importation and transmission potential. Simulation will allow for the testing of different control strategies or scenarios in a population into which malaria is sporadically imported by varying the levels of several important drivers in different combinations and examining their impact.

Post-elimination planning should consider at least three specific scenarios: first, control measures like ITNs or IRS are permanently maintained so as to ensure low levels of R_C _and thus defend against importation; second, surveillance and outbreak control capacity are strengthened while scaling back vector control to achieve cost-savings [6]; and third, depending on the geographical context, border screening or reduction of importation at its source is implemented to decrease the demands on other systems. For outbreak control, an important quantity is the fraction of all infections recognized by passive case detection and promptly treated with effective medication required to maintain elimination. It is also important to develop and simulate protocols about active or reactive case detection to estimate the number of teams that screen around high-risk areas or observed infections required to ensure sufficiently low risk of resurgence. These analyses will yield quantitative measures of how elimination may be maintained with different combinations of active and passive case detection and transmission- and importation-lowering activities. Over time, it is expected that these methods will be refined and increasingly linked to the operational and financial feasibility components, allowing direct estimation of the minimal and most cost-effective combination of interventions that can prevent reemergence.

### Operational feasibility

Operational feasibility has been defined as "an estimate of the applicability, in time and space, of technical methods under existing geographical, social, and climatic conditions," and "a government's potential to develop the organizational structure required to carry out a national anti-malaria programme" [54]. It describes whether, given the realities of infrastructure, transportation, communication, and human behaviour, "it is possible to create a national organization which will carry out the programme" [25] required to attain and sustain elimination as defined by the technical feasibility assessment. The operational feasibility assessment must translate the effective coverage levels prescribed by the technical assessment into real-world terms, a realistic mix of activities that meet or exceed the benchmarks described by the technical analysis. Such activities must be within the capabilities of the region's malaria programme and health infrastructure. In doing so, an operational feasibility assessment goes beyond a simple yes/no answer to the question of whether technical requirements can be achieved to provide a clear statement of how they will be operationalized. A first step to assess operational feasibility is thus to describe key factors that will influence whether technical thresholds for achieving and sustaining elimination can be achieved in a particular context. Secondly, potential barriers that must be overcome to enable scale-up to required levels need to be discussed. Lastly, an operational feasibility assessment should discuss qualitative issues related to ensuring a sufficiently supportive operational environment [18].

#### Is it operationally possible for elimination to be achieved?

The technical feasibility assessment defines the minimum fraction of the population that must be effectively protected from infection to achieve a value of R_C _that leads to elimination. To assess the operational feasibility of achieving these effective coverage levels, both the characteristics of the transmission-reducing tools used and the capacity of the malaria programme and the health system must be considered. These factors do not need to be perfect, but they should meet minimum requirements to provide the area under consideration and its malaria programme with the opportunity to meet required technical thresholds.

First, a realistic measure must be made of the effective coverage that can be achieved within the population of interest. Surveys of 13 sub-Saharan African countries in 2008 found median household ownership of ITNs of 56%, with a wide range of 8%-82% [1]. Achieving high levels of effective coverage by an intervention like ITNs is made more challenging in very rural regions with poor transportation infrastructure. Net coverage is highest where nets can be distributed for free through mass campaigns [55], so a programme's ability to mount such campaigns may be an important determinant of realistic effective coverage rates. An individual is effectively protected if there is no chance that transmission will occur. The effectiveness of an ITN, for example, will depend upon how often individuals actually sleep under the nets, whether the nets develop holes, and if the insecticide remains effective (over time, the protective effect may decrease as the result of weakened insecticide effectiveness and/or resistance even if overall coverage levels remain unchanged). While achieving high population coverage might be relatively easy to achieve, ensuring high usage levels has, according to data from the WHO Malaria World Report, been more challenging [1]. The fraction of the population covered by interventions is multiplied by the probability of the intervention being effective to yield the effective coverage of that intervention. Taking these factors and the degree of success of previous distribution campaigns into account, a realistic maximum effective coverage by each intervention should be estimated.

The fraction of the population actually protected by control measures is the relevant measure that should be compared directly with the required effective coverage levels described by the technical assessment to determine the operationally feasibility of those levels. In places where vectors are unaffected by ITNs, other strategies must be considered. Given a maximum current household ownership of ITNs of 82% in sub-Saharan African countries where data is available, and assuming that an ITN is used and maintained appropriately in 80% of cases, effective coverage is at most 66% in these countries. True effective coverage may be significantly lower, since household ownership reflects only that at least one net is owned and does not mean that all individuals in that household own nets. In certain areas or difficult to reach populations with low usage levels it will, therefore, be operationally challenging to achieve the effective coverage levels estimated to achieve elimination. In addition, a recent top-up campaign in Botswana demonstrated that even with strong IEC/BCC, additional gains, both in terms of coverage and usage, after the initial successful scale-up are comparatively much harder to achieve (A Tatarsky, personal communication). Sufficiently high effective coverage levels by nets alone can thus be operationally infeasible. However, sufficient effective coverage levels may still be achievable with combinations of measures, like IRS with ITNs.

#### Is it operationally possible to prevent reemergence following elimination?

The technical feasibility assessment produces a set of scenarios under which elimination may be sustained over time. Such scenarios can vary key drivers including maintenance of vector control measures, levels of surveillance, and importation-reducing activities. The operational assessment must determine which, if any, of these scenarios is achievable, taking into account the operational capacity of the malaria programme and the health system in general. The operational assessment should differentiate scenarios that are operationally challenging and politically unattractive from those deemed completely infeasible.

A post-elimination scenario involving maintenance of net coverage in perpetuity may prove to be a technically viable solution to avoiding resurgence, but an operational assessment would need to weigh the benefits - obviating the need for any importation-reducing activities or, potentially, greatly strengthened surveillance - against the operational challenges: a need for continual distribution campaigns, fatigue on the part of politicians and the populace once malaria is no longer an observable public health problem, the probability of eventual resistance to insecticides, and the lack of a perception that the country is malaria-free due to the continued use of protective measures.

Evaluating the operational feasibility of scenarios in which coverage by transmission-lowering interventions is scaled back involves assessing whether the surveillance system can be sufficiently strengthened to compensate. A perfect surveillance system that promptly detects and eliminates all new infections would in theory suffice to avoid resurgence regardless of the prevailing transmission potential. However, case detection methods have inherent limitations in detecting infections, especially asymptomatic infections [56].

The proportion of infections passively detected depends mainly on health seeking behavior, testing rates at the visited facility, and the sensitivity of the test used [57]. Each of these factors thus must be assessed in turn. Population-based surveys of treatment seeking behaviors should be used to evaluate whether geographical, financial or social barriers to diagnosis and treatment exist that would limit the capacity of the health system to pick up and eliminate new infections before they can cause onward transmission, thereby creating foci of infection where elimination will either never be achieved or easily reemerge. For a disease that will be extremely rare, it will be essential to keep the population informed on the importance of prompt health-seeking behaviour for all fevers and, where necessary, the continuous use for preventive measures. In addition the community can actively participate in surveillance activities for elimination [58-61] and the operational feasibility will, therefore, be influenced by the level of involvement by the local population and the IEC/BCC activities required to keep them informed and motivated. Minimum levels of human resources, training, and supply management will be required to ensure sufficient testing rates. While it is unlikely that elimination can be achieved nor maintained in settings where the health system is totally failing, health systems do not need to be perfect to reach effective levels of case detection and treatment.

It may be operationally difficult to influence some of these factors, especially in settings with a weak overall health system. In addition, the technical assessment may reveal that even very high levels of passive case detection (PCD) might not be sufficient to maintain elimination in places where potential transmission or importation of infections is high. In these cases, achieving required case detection levels will likely necessitate complementing PCD with some form of active case detection. Depending on the context, reactive screening around an index case, screening of high risk groups, or border screening might be most appropriate [57], but determining the feasibility of these strategies requires evaluating the availability of intensive human resources and other implementation challenges, including the need for robust reporting systems.

If achieving the necessary levels of surveillance is deemed operationally infeasible, it may be possible to offset these requirements by reducing the importation rate. Again, the operational feasibility of such an approach will vary by context; border screening, for example, will be a viable option for island settings where entry points are few and often controlled but operationally infeasible in countries with long porous borders. Cross-border initiatives may prove a more effective means of reducing importation at its source for such countries [23].

Regardless of the scenario, it is essential to verify the strength and operational capacity of the malaria programme. For most programmes this will require a deep human resource pool and the ability to shift competencies towards a stronger emphasis on surveillance and epidemiology skills. An empowered programme with flexible resources that can mount a swift response when cases are reported will be a key factor of any elimination attempt's operational feasibility. Some of the activities necessary to reduce transmission and avoid resurgence will also benefit from an enabling legal environment. Legislation can be used to impose compliance with certain essential activities on both individuals and businesses, though enforcement should be a last resort. Any elimination effort will benefit from a clear legal framework especially for activities such as blood screening or the compulsory spraying of private dwellings that could potentially be an infringement on individual privacy or a population's human rights [19].

### Financial feasibility

If sustained resources were limitless, elimination would be a universal goal. However, most malaria endemic regions face funding constraints, donor instability, insufficient human resources, and competing pressing health priorities. The economic value of malaria elimination relative to other strategies thus becomes open to discussion. Determining the feasibility and desirability of an elimination programme requires a thorough understanding of how much elimination will cost relative to alternative options, such as attempting to maintain CLM [47]. The financial component of the feasibility assessment examines these questions through analysis of the estimated costs of an elimination programme under a variety of scenarios and exploration of the challenges and potential solutions involved in sustainably securing the required finances.

There are three principal financial questions of interest to policymakers when contemplating an elimination programme. First, how much would the programme cost over time? Second, can sufficient funds be made available? And third, is this programme a cost-effective use of resources compared to other health and social sector priorities? The ideal information to provide to policymakers to answer these questions and guide their decisions would be robust comparative cost-benefit analysis [24] for both elimination and the alternative of CLM [47]. However, the challenges of such an exercise are "fraught with difficulties" [17]. The benefits of malaria elimination beyond its direct effect on morbidity and mortality are broad-reaching and difficult to enumerate or quantify; eliminating malaria may have positive effects on tourism, education, workforce productivity, health system reach, and performance and foreign investment, but a reliable estimation of these myriad effects and comparison with their values under the alternative of a continued control programme is a major challenge. In the likely event that it is not possible to conduct such a comprehensive analysis, it should focus on a robust financial comparison of elimination and CLM accompanied by qualitative judgments of the potential non-health benefits generated by elimination.

To do so, it is necessary to compare the potential cost of the programme if the goal is control with the alternative expenditure required to achieve and sustain elimination. Because elimination will be more expensive in the short-term, this comparison should take a medium- to long-term view to present an accurate picture of cumulative and annual costs over time, as determined to be policy-relevant by local decision-makers. Most current planning occurs on five-year budgeting cycles [40], so an appropriate long-term cost estimate for a control programme will need to be devised. Ideally, this estimate should represent an optimized control package of interventions rather than one that simply seeks to achieve universal access with all available tools, since the relative attractiveness of elimination will differ depending upon the cost of its alternative. In the absence of such an optimized cost estimate, it may be assumed less robustly that the currently budgeted costs of maintaining CLM (for example, from recent Global Fund proposals) will remain relatively stable over time and, assuming that drugs and insecticides remain effective, are mainly influenced by population growth. Analyses of recent or contemporary elimination programs have found that the average annual cost of achieving elimination is substantially greater than that of CLM and that elimination programs are unlikely to generate cumulative financial savings [23,24]. However, these analyses also found considerable variation in costs between country contexts, emphasizing the importance of conducting a robust financial analysis to assess elimination feasibility.

The costs of achieving the required intervention effective coverage levels to reach elimination and the necessary package of interventions for the scenarios that describe sustaining it thereafter should be determined according to the associated operational requirements. As with the budget for CLM, it is important to ensure a comprehensive set of costs covering such categories as personnel, consumables, equipment, travel, and training. Both budgets must be consistent in how they incorporate costs that may be shared by the general public health system; for example, ensuring access to health facilities may be required, but such an activity is not malaria-specific and thus may be excluded from or only partially accounted in the programme budgets.

Approximating costs for scaling up activities to required levels may prove challenging, especially since little empirical evidence may be available to understand how marginal costs may increase with coverage (increasing coverage of an intervention will likely require a greater outlay of resources to move from 90% to 95% than from 20% to 25%) and how the costs of different interventions interact with one another (i.e., if there are synergies that generate cost efficiencies). Additionally, considerable uncertainty is likely to exist around costs required post-elimination. Predicting how factors like importation or intrinsic transmission potential may change over the next several decades is not possible with any degree of certainty, meaning that the level and combination of interventions required to sustain elimination may be very uncertain. It is important to capture as much of this uncertainty as possible through scenario analyses, with corresponding examination of whether qualitatively different conclusions are reached about the relative annual and cumulative costs of elimination and control. If elimination is not cumulatively cost-saving over the time horizon of interest in all possible scenarios, it will be important for decision-makers to judge whether the cost increase is outweighed by other expected benefits.

Financial feasibility will not only depend on the additional cost of elimination compared to the cost of maintaining CLM, but also on the total magnitude of costs and the region's ability and willingness to cover those cost. Sufficient financing must be available to pay for the programme over the entire time horizon. Accordingly, the financial feasibility assessment should determine the risk of financial volatility in terms of the degree of donor dependence and the length of funding commitments, while considering whether the dependability of these funding sources would differ for an elimination or control programme. It is also important to assess the magnitude of governmental contributions, including how large a fraction of overall government spending and overall health spending would be comprised by the elimination programme. If this examination reveals a high risk of volatility, it will be necessary to examine whether mechanisms can be put in place to mitigate this risk and allow for continuous funding of malaria programmes even in the absence of the disease. Mechanisms and policies to increase the predictability and sustainability of global health funding, such as endowment funds or donor guarantee facilities, have been frequently explored, but there are few examples of successful applications for large-scale programs [62]. There are more cases of domestic governments taking steps to ensure sustained funding for certain health issues. The US government, for example, has employed targeted taxes to ensure sufficient ongoing resources to support those affected by immunization adverse events and "black lung" disease [63,64]. Financing solutions, however, are heavily dependent on the political and economic context and the health issue and so will need to be analyzed and developed distinctly in each region.

## Conclusion

The framework presented here represents a flexible, evidence-driven process for assessing strategic options and allocating limited resources efficiently once scale-up of malaria control has been achieved. It has been applied to help inform the decision-making process in Zanzibar [19], and future adaptation will continue to refine and improve these methodologies as they are applied in other regions. Because discussions of elimination today involve a concept very different from the 3-5 year time-limited campaign of GMEP, strategies must be planned as long-term, sustainable endeavors rather than quick outlays of resources. A nuanced approach to technical, operational, and financial feasibility is needed to account for the complexity of the system under consideration; an assessment that states elimination is impossible due to weakness in a single component - for example, a weak health system, a historically high level of endemicity, or a lack of precedent in similar regions - may miss offsetting strengths or advantages. In turn, a finding that elimination could be theoretically achieved in an area with current tools may fail to account for insurmountable operational obstacles or unrealistically high costs. A careful planning process that has considered not only whether elimination can technically be accomplished and sustained, but also whether it is operationally plausible and financially supportable over many years is thus of the utmost importance.

While existing guidance for assessing the overall feasibility of elimination is qualitative in nature [18], this framework provides a quantitative approach that forms the basis of the technical assessment and at the same time complements and informs the more qualitative components of the operational assessment. Specific, quantifiable metrics like the rate at which infections are imported into the region, the probability of each imported infection leading to onwards transmission, and the additional monetary costs of elimination over control are essential to allow rigorous assessment of the feasibility of these endeavours. Although exact feasibility thresholds for each of these metrics will need updating as this framework is refined, they permit a evidence-based means of evaluating the amount of effort required to achieve and maintain elimination. They also provide a quantitative basis for elimination scenario planning that can be used to determine the ideal mix of activities required to achieve and maintain elimination.

Following the admission that GMEP was unlikely to succeed in countries with weak public health infrastructures and highly endemic malaria in the early 1970s, support for the campaign quickly unraveled. Countries today may be tempted to declare the laudable goal of eliminating malaria from their borders, but they must bear in mind the perils of failing to achieve such promises. Rather than encouraging politically expedient, short-term elimination attempts, the global malaria community must foster an approach in which all countries recognize the importance of deliberate, careful evaluation and planning. Malaria elimination is a long-term enterprise, and robust strategic planning will be vital to permit it to succeed.

## Competing interests

BM, JMC, JC, and OS work within or assist the malaria control programme at the Clinton Health Access Initiative, which is supporting malaria elimination in southern Africa. BM, OS, SIH, and DLS serve as members of the Malaria Elimination Group. AJT declares that he has no competing interests.

## Authors' contributions

BM and SIH conceived of this framework. DLS, JMC, AJT, JC, OS and BM developed the methodology. BM, JMC and DLS drafted the manuscript. All authors read and approved the final manuscript.

## Appendix 1. Definitions of key terms

**Eradication **refers to the permanent reduction to zero of the worldwide incidence of infection [18].

**Elimination **refers to a state where interventions have interrupted endemic transmission and limited onward transmission from imported infections below a threshold at which risk of reestablishment is minimized [47].

**Controlled low-endemic malaria **refers to a state where interventions have reduced endemic malaria transmission to such low levels that it does not constitute a major public health burden, but at which transmission would continue to occur even in the absence of importation [47].

**Parasite Rate (PR) **refers to the proportion of the population found to carry asexual blood-stage parasites [65].

**Entomological Inoculate Rate (EIR) **refers to the rate at which people are bitten by infectious mosquitoes [66].

**Transmission potential **refers to the propensity for malaria parasites to spread through the population; or in quantitative terms, the number of incident malaria cases that would arise from each malaria case within a defined area or focus [26].

**Intrinsic transmission potential **refers to the maximum number of incident malaria cases per case that would occur in the absence of all control measures and immunity [47].

**Importation rate **refers to the probability of malaria reintroduction based on an area's proximity to other malarious areas and the movement into that area of infected humans or infected *Anopheles *mosquitoes; or in quantitative terms, the number of malaria infections per 1,000 persons per year that did not originate within the area of interest [26].

**Effective coverage **refers to the fraction of the population that is fully protected by a malaria intervention; it is thus distinct from "coverage" in the general sense, which refers only to the distribution of control measures but not necessarily whether they will successfully prevent malaria transmission in every case. For example, 100% net coverage will correspond to a lower effective coverage assuming some fraction of nets are improperly treated, contain holes, or are not slept under every night [35].
